# Analysis of the research progress on the deposition and drift of spray droplets by plant protection UAVs

**DOI:** 10.1038/s41598-023-40556-0

**Published:** 2023-09-11

**Authors:** Qin Weicai, Chen Panyang

**Affiliations:** 1 Suzhou Polytechnic Institute of Agriculture, Suzhou, 215008 China; 2 Nanjing Institute of Agricultural Mechanization, Ministry of Agriculture and Rural Affairs, Nanjing, 210014 China; 3 Nanjing Institute of Technology, Nanjing, 211167 China

**Keywords:** Ecology, Plant sciences

## Abstract

Plant protection unmanned aerial vehicles (UAVs), which are highly adapted to terrain and capable of efficient low-altitude spraying, will be extensively used in agricultural production. In this paper, single or several independent factors influencing the deposition characteristics of droplets sprayed by plant protection UAVs, as well as the experimental methods and related mathematical analysis models used to study droplet deposition and drift, are systematically investigated. A research method based on farmland environmental factors is proposed to simulate the deposition and drift characteristics of spray droplets. Moreover, the impacts of multiple factors on the droplet deposition characteristics are further studied by using an indoor simulation test system for the spraying flow field of plant protection UAVs to simulate the plant protection UAVs spraying flow field, temperature, humidity and natural wind. By integrating the operation parameters, environmental conditions, crop canopy characteristics and rotor airflow, the main effects and interactive effects of the factors influencing the deposition of spray droplets can be explored. A mathematical model that can reflect the internal relations of multiple factors and evaluate and analyze the droplet deposition characteristics is established. A scientific and effective method for determining the optimal spray droplet deposition is also proposed. In addition, this research method can provide a necessary scientific basis for the formulation of operating standards for plant protection UAVs, inspection and evaluation of operating tools at the same scale, and the improvement and upgrading of spraying systems.

## Introduction

**Figure 1:**
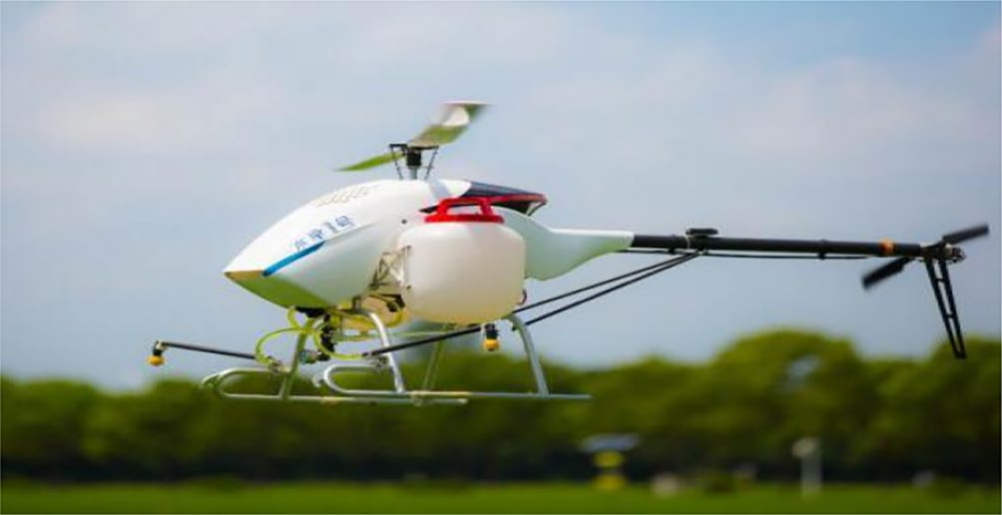
Single-rotor UAV spraying.

**Figure 2:**
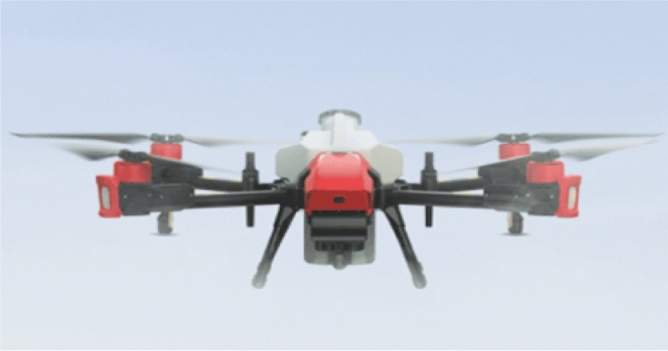
Multirotor UAV spraying.

In agriculture, aerial spray is widely used to spray fertilizers, herbicides, fungicides and other materials used for crop protection^1^. Compared with large fixed-wing agricultural aircraft, small unmanned aerial vehicles (UAVs) are particularly advantageous because they are highly maneuverable and do not need any airport for taking off or landing^2^. In recent years, aerial machinery for plant protection, especially aerial spray by small plant protection UAVs, has developed rapidly^3^. Small plant protection UAVs have greater application prospects in agricultural production because of their better terrain adaptability and low-altitude spraying capability (Figs. 1 and 2)^4–7^. However, as an emerging technology, UAV spraying technology in agricultural pest control are not common due to the lack of operational standards and uncertainty about the best spraying parameters, which leads to a series of problems, such as the poor uniformity of droplet deposition distribution and low levels of fog deposition.

Some studies have shown that if the aerial spraying parameters are not set scientifically, it will lead to not only repeated spraying and missed spraying, degrading the effect of pest control but also pesticide drift^8^. The use of new pesticide additives and the innovative research and development of precise spraying equipment of plant protection UAVs along with its safe and efficient use in the prevention and control of diseases, pests and weeds are indispensable means to increase the pesticide deposition amount and reduce drift. Studying the deposition characteristics of spray droplets is not only of scientific significance for the development of new pesticide formulations and precise spraying equipment of plant protection UAVs but also of practical guiding significance for the safe and efficient use of pesticides in farmland. Due to many factors, such as the natural environment, pesticide characteristics, crop canopy characteristics, and plant protection UAV operating parameters, it is a complicated process to study the uniformity and penetration of spray droplets. To improve the spraying effect and reduce drift, scientific and technological staff all over the world have carried out a large number of exploratory studies on the deposition and drift characteristics of spray droplets through field or wind tunnel experiments or mathematical model analysis^9–13^. The main factors and secondary factors influencing the characteristics of droplet deposition and drift are organized from the many influencing factors (nozzle, droplet, aircraft type, weather factors, etc.), and the functional relationship between the amount of different droplet deposition and drift and their influencing factors are determined. However, there are not sufficient deposition models for plant protection rotor UAVs, and the existing models consider only a few influencing factors, which need to be further modified.

With the development of UAV technology, there are an increasing number of studies on the droplet deposition rules, operation parameter optimization and evaluation methods of pesticides applied by plant protection UAVs in rice fields and maize fields^14–17^; however, these studies have defects in that the meteorological factors in the farmland environment are unstable and uncontrollable, the UAV track easily deviates, resulting in the poor uniformity of droplet deposition distribution (the coefficient of variation may be above 40%^16^, while it is usually below 10% for spraying by ground equipment), the test result cannot be well repeated, and different types of UAVs cannot be easily evaluated at the same scale. Thus, it is difficult to evaluate the droplet deposition characteristics of different types of UAVs scientifically. Some research has established mathematical models to study the impact of plant protection UAV operating parameters (operating height, operating speed, and spraying flow rate) on droplet deposition and drift characteristics^18–20^ and determined the main effects influencing droplet deposition. However, due to the lack of conformity between the assumptions of these models and farmland practice, they neglected the influence of the characteristics of the crop canopy and the interaction of multiple factors such as the environment, crops, and operating parameters of application equipment on the droplet deposition characteristics (uniformity of distribution and penetration), making the results obtained through analysis with existing mathematical models highly deviate from practice.

In this paper, the current status and problems of research on the deposition and drift of spray droplets from plant protection drones are introduced, and the importance of research in this area to improve the effectiveness of pesticide application and reduce drift hazards is emphasized. The need for more in depth, comprehensive and systematic research on the deposition and drift of spray droplets from plant protection drones is highlighted, and the problems and challenges of the current research are pointed out, providing important guidance and references for future research.

## Research on the influencing factors of spray droplet deposition characteristics

Studying droplet deposition characteristics (uniformity and penetration) is always a major subject in pesticide application technology research^21^. The deposition characteristics of spray droplets are influenced by application techniques and equipment, crops, the environment, etc. Detailed influencing factors include the wind speed, wind direction, leaf area index, target crop canopy structure, leaf inclination, leaf surface characteristics, and characteristics of the spray droplet population (release height, release rate, application liquid volume, spray droplet particle size spectrum)^22–24^.

Several studies have investigated the influence of various factors on droplet deposition characteristics in plant protection UAV spraying. Diepenbrock noted that plant leaf characteristics, such as size, inclination angle, drooping degree, and spatial arrangement, impact the composition quantity and distribution quality within the crop canopy structure, subsequently affecting droplet penetration and deposition^25^. Song et al. found that altering the initial velocity of droplets increases deposition amounts on horizontal and vertical targets. Factors like flying altitude and speed of different aircraft types have been extensively studied for their influence on droplet deposition and drift^26^. Qiu et al. used an orthogonal experimental method to study the deposition distribution rules of droplets sprayed by unmanned helicopters at different flying heights and speeds under field conditions. They established a relationship model that clarifies the interactions between deposition concentration, uniformity, flying speed, and flying height, providing valuable insights for optimizing spray operation parameters^18^. Chen et al. investigated the pattern of aerial spray droplet deposition in the rice canopy using a small unmanned helicopter. They explored the effects of different spraying parameters on droplet distribution, specifically analyzing the deposition of growth regulator spraying^27^. Wang et al. proposed a method for testing the spatial mass balance of UAV-applied droplets and conducted field experiments on three types of UAVs to accurately determine the spatial distribution of the droplets and the downdraft field. They also conducted an experimental study on the droplet deposition pattern of hovering UAV variable spraying and highlighted the significant impact of downward swirling airflow on droplet deposition distribution^14^. Qin et al. focused on the influence of spraying parameters, such as operation height and velocity, of the UAV on droplet deposition on the rice canopy and protection efficacy against plant hoppers, using water-sensitive paper to collect droplets and statistically analyzing their coverage rates. The findings indicated that UAV spraying exhibited a low-volume and highly concentrated spray pattern^19^.

**Figure 3:**
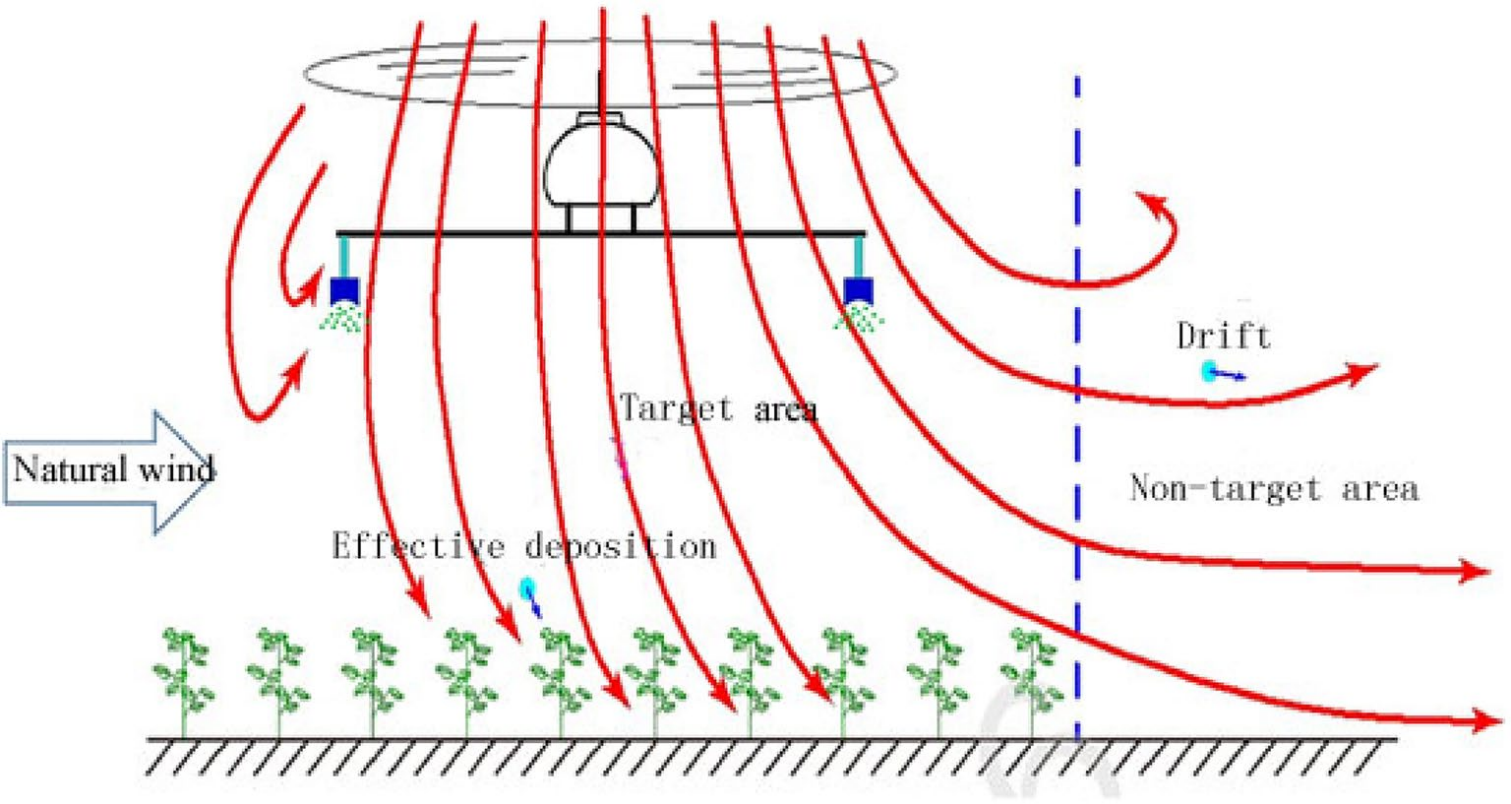
Description of the deposition and drift with rotor UAV spraying.

In summary, there are many factors influencing the deposition characteristics (uniformity and penetration) of spray droplets. However, in most of the current research on spraying by plant protection UAVs, only the influence of factors such as the flying height and flying speed on droplet deposition in the field environment is taken into consideration. Considering the influence of the interaction between environmental factors, crop canopy characteristics (growth stage, leaf area index, leaf inclination angle) and plant protection UAV spraying parameters on droplet deposition characteristics, there is neither in-depth understanding nor relevant reports, especially under controllable environmental conditions (Fig. 3). To promote high-efficiency spraying technology for plant protection UAVs, targeted basic research should be carried out on the analysis of the influencing factors of plant protection UAV spraying and the optimal deposition of droplets.

## Research on the experimental means and testing methods of droplet deposition and drift

At present, the deposition and drift of droplets are mainly researched by field tests and wind tunnel tests^28–32^. Field test research on pesticide deposition and drift is similar to the actual situation, but it is quite difficult to acquire correct data due to the constant changes in meteorological factors such as the wind speed, wind direction, temperature and humidity. In addition, Emilia et al. noted that the terrain and plant morphology also influence the wind flow and droplet deposition, leading to considerable deviation among repeated test results^33^. Therefore, it is difficult to accurately determine the total amount and distribution of pesticides drifting in the air^34^. The wind tunnel laboratory can provide a controllable environment to simulate the external spraying conditions, and the wind speed and direction can be easily controlled. Therefore, it is an important means to study the drift characteristics of spraying components and avoid many defects in field test research^10,35^. The typical wind tunnels that are widely used in agricultural aviation spraying technology are shown in Table 1^36,37^.

**Table 1:** Typical wind tunnel and parameters used for agricultural aviation spray.

No	Name	Speed/(m · s^−1^)	The test section size/m (width × height × length)	Architectural feature	Main application	Institution
1	Silsoe	0–15	4 × 2 × 5	Eiffel type	Spray drift and nozzle structure features	SRI
2	LSWT	0.2–6.7	1.2 × 1.2 × 14.6	Direct current closed	Spray drift, droplets bead spectrum and nozzle structure; DRT technology	USDA-ARS
3	HSWT	Up to 90	0.3 × 0.3 (open type, without fixed aspect ratio)	Direct current closed	Spray drift, droplets bead spectrum and nozzle structure; DRT technology	
4	ATB	0–20	3 × 2 × 20	Boundary layer wind tunnel with rotating platform	Complex landform droplets drift, poultry breeding cycle ventilation facilities	Germany′s leibniz institute of agricultural engineering
5	–	0–5	2 × 2 × 6	Turbulence wind tunnel	Freon, spray drift, Droplets bead spectrum	Belgium Gembloux AgroBioTech Agricultural University
6	–	Up to 83	1.75 × 1 × 10	Air conditioning open circuit closed	Spray drift, Droplets bead spectrum, Nozzle classification	CPAS
7	JKI(BBA)	0.3–15	2.5 × 1.6 × 10	Air conditioning backflow closed	Members of the European Union for performance test of spraying parts and machinery anti floating contrast grading	JKI(BBA)
8	NJS-1	1.0–10	1.2 × 1.8 × 7.5	Direct current closed	The performance testing of spray plant protection machinery	Nanjing Agricultural Mechanization Institution

**Figure 4:**
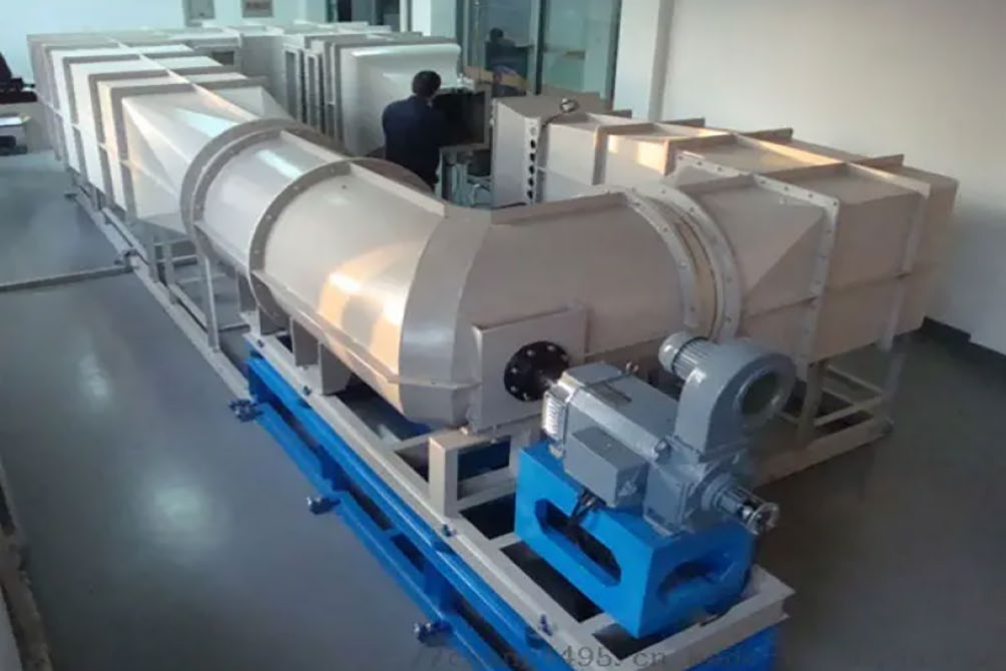
Circle closed low-speed wind tunnel.

**Figure 5:**
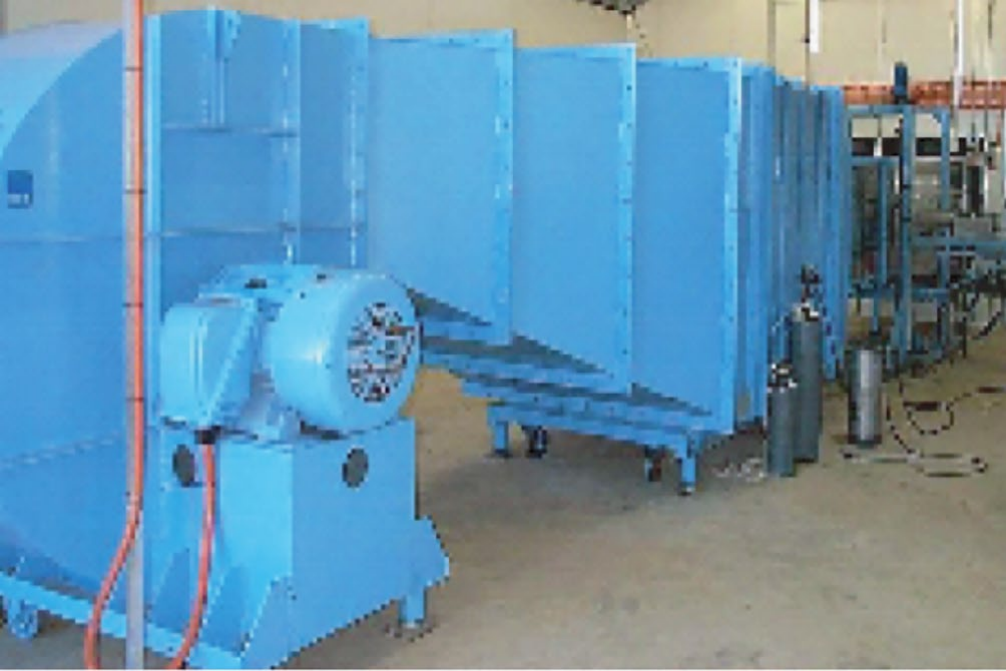
Open wind tunnel.

**Figure 6:**
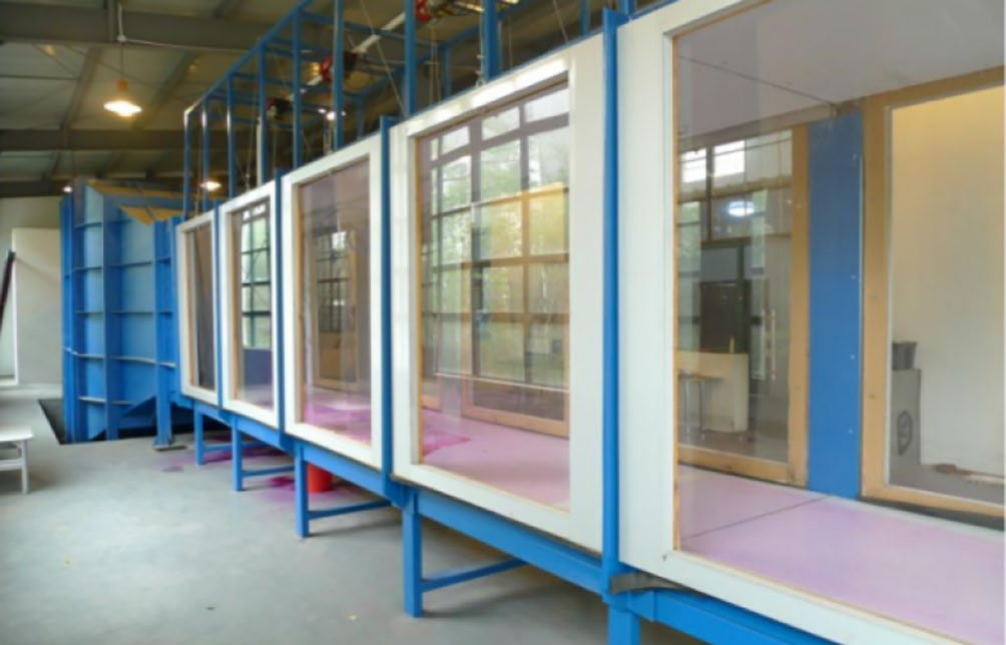
NJS-1DC closed wind tunnel.

**Figure 7:**
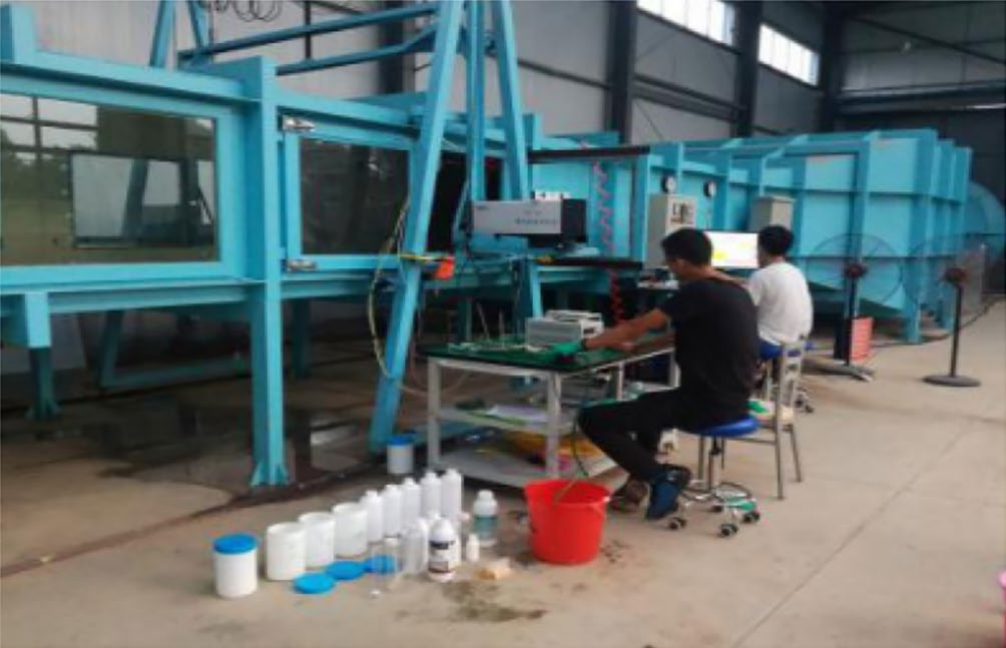
High and close speed composite wind.

Internationally well-known professional research institutions for pesticide application, such as the Julius Kuehn Institute-Federal Research Centre for Cultivated Plants (JKI, formerly BBA) and USDA-Agricultural Research Service, Application Technology Research Unit (USDA-ARS-ATRU), have a circular closed low-speed standard wind tunnel (Fig. 4). This wind tunnels are widely used to assess the distribution, degradation and drift of pesticide sprays, simulating real crop and environmental conditions. The advantages are that they provide accurate measurements of pesticide distribution and drift and are able to reproduce wind field conditions in realistic environments. However, circular low-speed wind tunnels have limitations when it comes to parameters such as spray particle size, density and flow rate for different pesticides. The Silsoe Research Institute, UK (SRI) has a standard linear low-speed wind tunnel. This wind tunnel can be used to test the performance of agricultural mechanised sprayers and the design of sprayers. The advantage is that they can simulate actual operating conditions and can accurately test the performance and flow rate of agricultural mechanised sprayers. However, linear low speed wind tunnels are typically more expensive than circular wind tunnels and can only simulate a single environmental condition. The Center for Pesticide Application and Safety (CPAS) of the University of Queensland in Australia has an open-path wind tunnel (Fig. 5). This type of wind tunnel can be used to test aspects such as drift and particle distribution of agricultural sprayers. The advantages are ease of operation, low cost and the ability to reproduce wind fields under different environmental conditions. However, open path wind tunnels do not simulate realistic crop environments and have unstable wind speeds. In 2014, the Nanjing Institute of Agricultural Mechanization, Ministry of Agriculture and Rural Affairs, built the NJS-1 plant protection direct flow closed wind tunnel (Fig. 6). This type of wind tunnel is mainly used to evaluate different sprayers in terms of performance and droplet distribution. The advantages are the ability to simulate a realistic farm environment with high accuracy and the ability to test different types and brands of sprayers. However, straight-through enclosed wind tunnels are only suitable for small equipment and small-scale trials and are costly. In 2018, the National Center for International Collaboration Research on Precision Agricultural Aviation Pesticide Spraying Technology of South China Agricultural University built a high- and low-speed composite wind tunnel for agricultural aviation research (Fig. 7). This wind tunnel is suitable for agricultural aerial research and can simulate the effects of spraying at different heights and wind speeds. The advantage is that it can accurately test the effects of pesticide spraying at different heights and speeds, and can improve the efficiency and accuracy of agricultural aerial spraying. However, high and low speed composite wind tunnels are relatively costly and require a high level of technology and equipment requirements. As the basic conditions for technical research, these wind tunnels have made great contributions to the study of pesticide deposition and drift rules, product testing, and product optimization^38–42^. However, for the study of spray droplet deposition and drift under the disturbance of the wind field of plant protection UAVs, the single-direction wind tunnel simulation test is still insufficient to simulate the combined effect of the downward swirl flow under the rotor and the natural wind. In addition, the existing agricultural wind tunnels are limited in size, so plant protection UAVs cannot be placed. In the military, a scaled model method is used to put UAVs into wind tunnels for research^43,44^, but it is not suitable for research on pesticide spraying with plant protection UAVs, and the airflow will rebound from the tunnel wall.

Another important test technique for drift research is the sampling and analysis of droplet drift. Test studies on the drift of aerial mist in developed countries such as the United States and Germany are carried out with advanced test instruments, including automatic air samplers, gas or liquid chromatography, fluorescence analyzers, and electronic scanners. to collect and analyze the droplet deposition amount, the number of droplets, the coverage density of droplets, and the content of substances and study the correlation between additive concentration, spraying height and drift^4,45,46^. However, these traditional methods involve a long collecting and processing cycle, samples have to be processed in the lab, and it is difficult to express the dynamics of droplets in air. Particle image velocimetry (PIV) and LIDAR scanning test methods can solve the above problems, and each has its own advantages. PIV can obtain the three-dimensional spatial velocity vector of droplets and droplet size with a high sampling accuracy but limited spatial measurement scale^47–49^; the LIDAR scanning method, realized by layered scanning, can quickly and accurately obtain the large-scale spatial droplet point cloud data and inversely form the three-dimensional distribution and temporal-spatial change of the droplets, but cannot reflect the spatial velocity vector change of the droplets^50^. The advantages, disadvantages and applications of droplet deposition and drift measurement methods are shown in Table 2^51^.

**Table 2:** Advantages, disadvantages and applications of various measurement methods.

Measurement method	Acquirable information	Advantage	Disadvantage	Application
Spot method	Droplet size	The principle is simple, and the original form of the droplets can be preserved	The operation is complicated and limited by the testing site	Indoor testing
Paper card method	Droplet size and coverage	Simple operation and intuitive result	The measurement accuracy is impacted by the diffusion coefficient of the test paper	To acquire the droplet size and coverage of representative areas
Cotton thread or nylon thread method	Droplet deposition amount	Tiny droplets can be captured	Data processing is complicated	Droplet drift
Stain method	Droplet deposition amount, droplet size and distribution	Low cost	Data processing is complicated, and the result accuracy is not high	Where accuracy is not much required
Fluorescent particle tracing method	Droplet deposition amount	High accuracy	High cost, droplet size and distribution cannot be acquired	Outdoor detection focusing on acquiring the deposition amount
Phase Doppler particle analysis	Droplet velocity, particle size, number of droplets per unit time	High measurement accuracy	Not suitable for the detection of large droplets	Droplet field with smaller droplet size
Laser/droplet image analyzer	Droplet size and moving speed	Real-time images of the droplet field can be acquired, high measurement accuracy	Not suitable for detection of droplet field with a high droplet density	Droplet field with low droplet density
Laser particle size analyzer	Cumulative distribution ratio of any droplet size to an interval between Dv0 and Dv100	High measurement accuracy	Only the droplet data on the laser line can be acquired	An indoor test to acquire the droplet size distribution

Overall, the sampling and analysis of droplet drift, along with techniques such as PIV and LIDAR scanning, play a crucial role in studying and understanding the behavior of droplets during aerial spraying. These methods provide valuable insights into droplet deposition, drift patterns, and the effects of various factors, enabling researchers to optimize spray practices, minimize drift, and enhance the efficiency and effectiveness of plant protection UAV applications.

## Research on the mathematical analysis model of spray droplet deposition characteristics

In the development of spraying equipment and the determination of the optimal deposition conditions for spray, a large amount of data and information are needed to explain the influence of different factors on the spraying performance and the relationship between variables. At present, spraying drift modeling can be divided into models based on mechanics and models based on statistics^52–54^.

One of the models based on mechanics analyzes the movement of a single droplet in the airflow field by the Lagrangian trajectory tracking analysis method. Teske et al. established the AGDISP model by the analytical Lagrangian method to describe aerial spraying under the condition of ignoring the influence of aircraft wake and atmospheric turbulence^46^. This model takes not only the aircraft type, environmental conditions, and droplet properties but also the influencing factors of the nozzle model into consideration. The user can input the parameters of the nozzle, droplet spectrum, aircraft type and weather factors. from an internal database and predict the drift potential. It can effectively and accurately predict a range of 20 km but is generally used for fixed-wing aircraft. Duga et al. and Gregorio et al. also studied the deposition distribution of aerial spray in orchards with the Lagrangian discrete phase model, and the result of the numerical model showed that the prediction error of total deposition on the fruit tree canopy is above 30%^48,51^. Dorr et al. developed a spray deposition model for whole plants based on L-studio, which takes into account the plant leaf wettability, impact angle, droplet break-up and rebound behavior, and the number of sub-droplets produced^55^. In 2020, Zabkiewicz et al. used an updated version of the software based on this model, developing a new user interface and refining the droplet fragmentation model^56^.

Another model based on mechanics is realized with the CFD (Computational Fluid Dynamics) method^57,58^, but there are still large errors between the simulated value and the real value of some models due to various factors. Holterman et al. carried out a series of cross-wind single nozzle field experiments in consideration of the traveling speed, entrained airflow, geometric parameters of the farmland, sprayer system setting parameters and environmental factors when studying the droplet deposition drift model of ground boom sprayers to calibrate the mathematical model. The results showed that when the height from the crop canopy is less than or equal to 0.7 m, the error between the test and the model simulation is within 10%, but the error between droplet deposition and drift prediction gradually increases as the height of the spray boom increases^59–61^.

**Figure 8:**
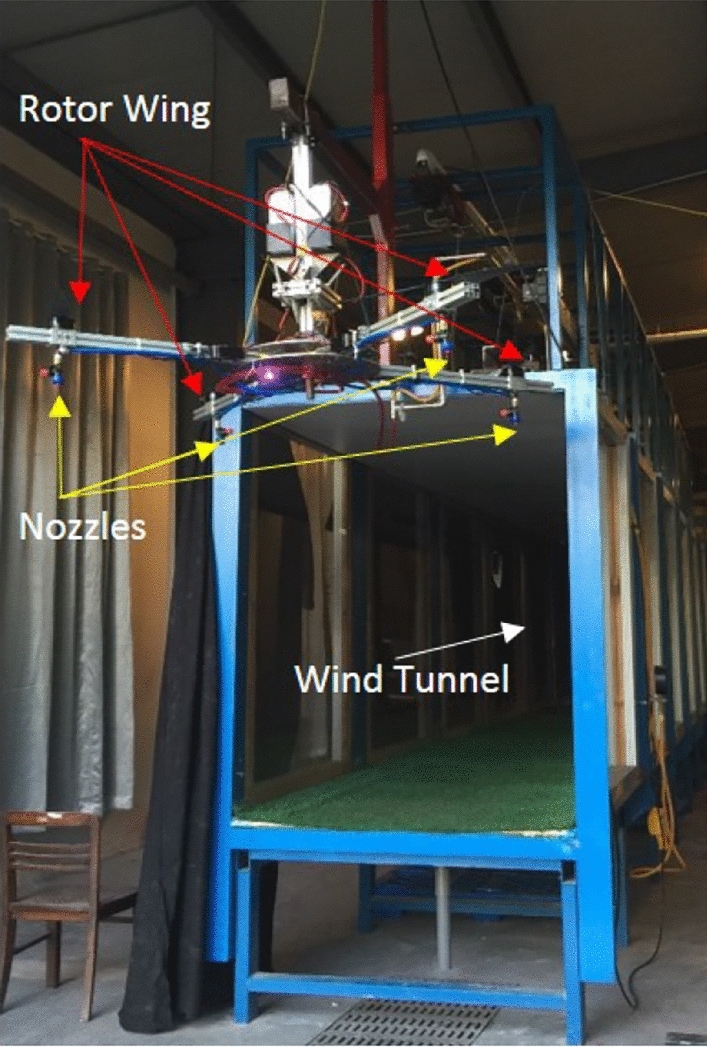
Rotor wind field test platform based on a wind tunnel.

**Figure 9:**
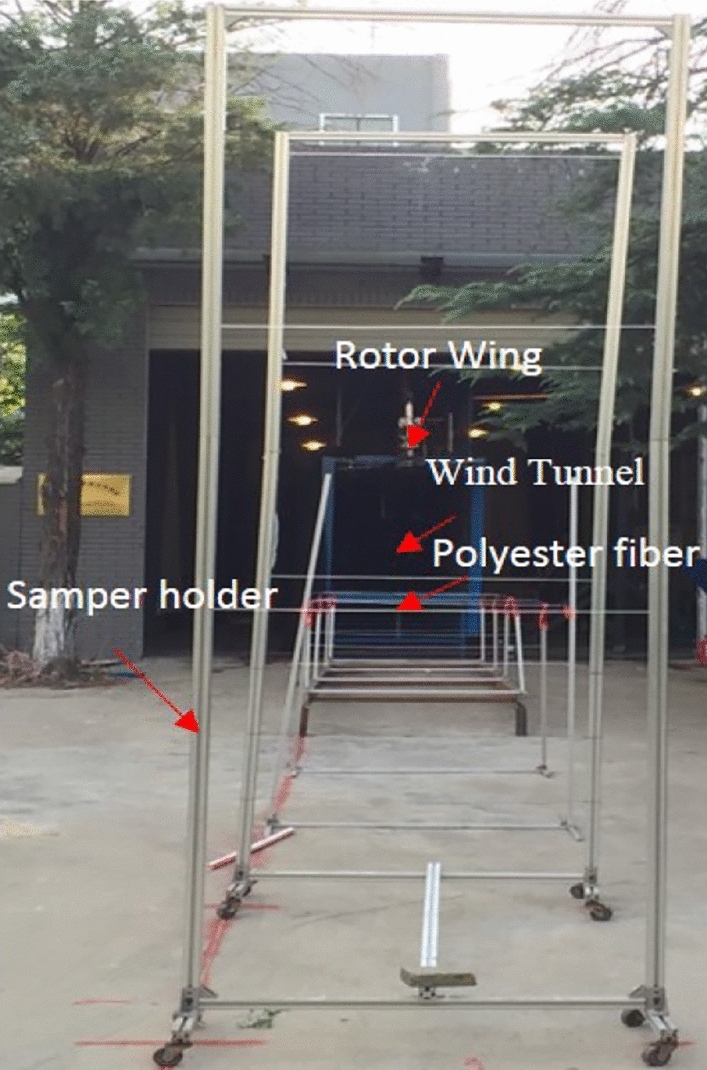
Layout scene of droplet drift.

Chinese scientific and technological staff have conducted experimental research and numerical analysis on the numerical simulation and mathematical modeling of spraying droplet deposition and drift prediction of ground plant protection equipment and have drawn some conclusions that physical quantities such as the operating speed, droplet size and crosswind impact the droplet deposition and drift process (Figs. 8 and 9)^62,63^. Zhu et al. developed the DRIFTSIM based on CFD and Lagrangian methods with a CFD simulation database for ground drift prediction and a user interface to access drift-related data^64^. Hong et al. constructed an integrated computational hydrodynamic model to predict the deposition and transport of pesticide sprays under the canopy in apple orchards during different growth periods^65^.

The above research proves that computer simulation technologies are widely applicable to the prediction research of droplet deposition under various complicated wind-supply airflow conditions^66^. The existing AGDISP model is relatively developed and only suitable for research on fixed-wing aircraft, which is very different from research on plant protection UAVs. The current plant protection UAV spraying prediction model still has problems such as large relative errors between the experimental value and simulation value of the deposition and drift at each measurement point. Therefore, the prediction accuracy of the numerical model for the spray droplet deposition of plant protection UAVs is still low and needs to be improved, and there is a lack of in-depth basic research on analyzing the rotor flow field and establishing mathematical analysis models for droplet deposition^67^.

## The rotor wind field test platform and droplet drift

The use of UAVs for crop spraying has become increasingly popular due to its efficiency and effectiveness. However, accurately analyzing the spraying process is challenging due to the complex flow field of the droplets in the air and the multitude of factors that can affect their deposition characteristics. Current testing systems rely on simple methods such as static targets or trays, which do not accurately represent the dynamic and complex nature of the real environment. To better study the UAV spraying flow field, a corresponding indoor simulation test system is needed. The indoor simulation system proposed in this study combines a natural wind simulation system and a rotor simulation system that can simulate several factors present in the natural environment that affect droplet deposition characteristics. The natural wind simulation system can effectively replicate wind speed variations, which is a key factor influencing droplet dispersion and deposition. By adjusting the settings of the wind simulation system, it is possible to replicate a range of wind speeds encountered in the field, allowing researchers to study the effects of different wind speeds on droplet behaviour and deposition. By adjusting the settings of the rotor simulation system, it is possible to demonstrate the magnitude of the downwash airflow at different speeds of the UAV rotor. However, it is important to note that while wind speed variations can be simulated, other factors, such as wind direction and turbulence, may have limitations in being accurately replicated in an indoor simulation system. These factors may require further development of simulation techniques to achieve more accurate replication. Nevertheless, the inclusion of natural wind simulation systems and rotor simulation systems in indoor simulation setups provides a valuable tool for studying the effects of wind speed.

The fluorescence tracer method involves adding a fluorescent dye or tracer to the liquid spray mixture used in the UAV spraying process. When these droplets containing the fluorescent tracer are released into the air, they can be illuminated with a specific wavelength of light, typically ultraviolet (UV) light. The fluorescent dye absorbs this UV light and re-emits it at a longer wavelength, usually in the visible range.

The high-speed camera is synchronized with the UV light source and captures the emitted fluorescent signals from the droplets. By analyzing the recorded video footage, researchers can precisely track the movement and behavior of the fluorescent droplets in the air. The high-speed camera captures images at a rapid frame rate, allowing for the visualization and analysis of the droplet flow field in detail.

**Figure 10:**
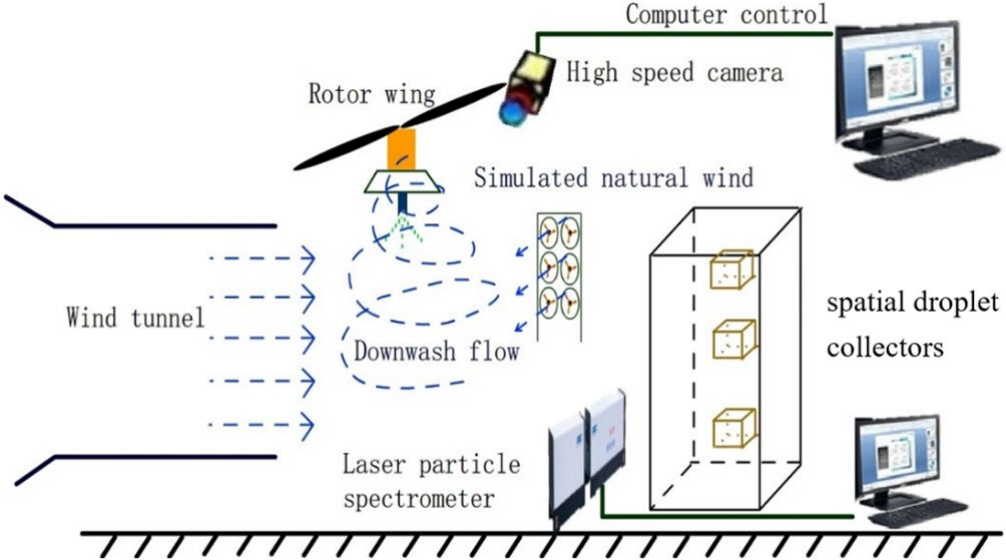
Diagram of the rotor wind field test platform and droplet drift.

The proposed indoor simulation test system for the spraying flow field of plant protection UAVs is a comprehensive and innovative method that combines the fluorescence tracer method and high-speed camera method to accurately track the dynamic changes in the local droplet flow field in the air. This system also includes a natural wind simulation system, which allows for the more realistic simulation of the actual environment, and thus more accurately reproduces the complex factors that affect droplet deposition characteristics. This method represents a significant improvement over existing testing systems, as it provides a more accurate and comprehensive analysis of the deposition process of droplets affected by multiple factors, enabling researchers to more effectively study the flow field and optimize the spraying process for plant protection UAVs. Overall, this proposed system has the potential to revolutionize the study of UAV spraying flow fields and could lead to significant advancements in the field of plant protection. Therefore, the method proposed in this paper is superior to the methods currently in use (Fig. 10).

## Conclusion

In conclusion, existing studies on plant protection UAV spraying have primarily focused on isolated factors, such as flying height, flying speed, and nozzle flow, without considering the interaction effects among other influential factors. This limitation calls for the need to conduct experimental research that combines spray droplet deposition characteristics with crop canopy characteristics in a controllable environment, encompassing environmental conditions and operating parameters. The proposed research aims to address this gap by developing an indoor simulation system that incorporates a natural wind simulation system. This innovative system enables the study of droplet deposition characteristics influenced by multiple factors in a realistic environment. By statistically analyzing the factors affecting droplet deposition and establishing a multivariable relationship model, optimal droplet deposition suitable for field operation decision-making of plant protection UAVs can be quantified and evaluated. This research presents an effective technical pathway for understanding the deposition patterns of droplets sprayed by plant protection UAVs and supports the formulation of relevant pesticide application standards for plant protection UAVs.

## Data Availability

The datasets used and/or analyzed during the current study are available from the corresponding author on reasonable request.
